# Rare Causes of Primary Adrenal Insufficiency: Genetic and Clinical Characterization of a Large Nationwide Cohort

**DOI:** 10.1210/jc.2015-3250

**Published:** 2015-11-02

**Authors:** Tulay Guran, Federica Buonocore, Nurcin Saka, Mehmet Nuri Ozbek, Zehra Aycan, Abdullah Bereket, Firdevs Bas, Sukran Darcan, Aysun Bideci, Ayla Guven, Korcan Demir, Aysehan Akinci, Muammer Buyukinan, Banu Kucukemre Aydin, Serap Turan, Sebahat Yilmaz Agladioglu, Zeynep Atay, Zehra Yavas Abali, Omer Tarim, Gonul Catli, Bilgin Yuksel, Teoman Akcay, Metin Yildiz, Samim Ozen, Esra Doger, Huseyin Demirbilek, Ahmet Ucar, Emregul Isik, Bayram Ozhan, Semih Bolu, Ilker Tolga Ozgen, Jenifer P. Suntharalingham, John C. Achermann

**Affiliations:** Department of Pediatric Endocrinology and Diabetes (T.G., Z.A., A.B., S.T.), Marmara University, Istanbul 34899, Turkey; Institute of Metabolism and Systems Research (T.G.), University of Birmingham, Birmingham B15 2TT, United Kingdom; Department of Genetics and Genomic Medicine (F.B., J.S., J.C.A.), University College London Institute of Child Health, University College London, London WC1N 1EH, United Kingdom; Department of Pediatric Endocrinology and Diabetes (N.S., F.B., B.K.A., Z.Y.A.), Istanbul Faculty of Medicine, Istanbul University, 34452 Istanbul, Turkey; Clinics of Pediatric Endocrinology (M.N.O., H.D.), Diyarbakir Children's Hospital, 21100 Diyarbakir, Turkey; Clinics of Pediatric Endocrinology (Z.A., S.Y.A.), Dr Sami Ulus Obstetrics and Gynecology, Children‘s Health and Disease Training and Research Hospital, 06100 Ankara, Turkey; Department of Pediatric Endocrinology and Diabetes (S.D., S.O.), Ege University, 35040 Izmir, Turkey; Department of Pediatric Endocrinology and Diabetes (A.B., E.D.), Gazi University, 06550 Ankara, Turkey; Pediatric Endocrinology Clinic (A.G., M.Y.), Goztepe Educational and Research Hospital, 34810 Istanbul, Turkey; Department of Pediatrics (A.G.), Amasya University Medical Faculty, 05189 Amasya, Turkey; Pediatric Endocrinology Clinic (K.D.), Dr Behçet Uz Children's Hospital, Izmir, Turkey; Department of Pediatric Endocrinology and Diabetes (A.A.), Inonu University, Malatya, Turkey; Clinics of Pediatric Endocrinology (M.B.), Konya Training and Research Hospital, 42100 Konya, Turkey; Department of Pediatric Endocrinology and Diabetes (O.T.), Uludag University, 16059 Bursa, Turkey; Department of Pediatric Endocrinology and Diabetes (G.C.), Eylul University, 35210 Izmir, Turkey; Department of Pediatric Endocrinology and Diabetes (B.Y.), Cukurova University, 01330 Adana, Turkey; Clinics of Pediatric Endocrinology (T.A.), Kanuni Sultan Suleyman Education and Research Hospital, 34303 Istanbul, Turkey; Pediatric Endocrinology Clinic (A.U.), Sanliurfa Children's Hospital, 63300 Sanliurfa, Turkey; Pediatric Endocrinology Clinic (E.I.), Gaziantep Children's Hospital, 27010 Gaziantep, Turkey; Department of Pediatric Endocrinology and Diabetes (B.O.), Faculty of Medicine, Pamukkale University, 20160 Denizli, Turkey; Department of Pediatric Endocrinology and Diabetes (S.B.), Faculty of Medicine, Duzce University, 81620 Duzce, Turkey; Department of Pediatric Endocrinology and Diabetes (I.T.O.), Bezm-i Alem Vakif University, 34093 Istanbul, Turkey

## Abstract

**Context:**

Primary adrenal insufficiency (PAI) is a life-threatening condition that is often due to monogenic causes in children. Although congenital adrenal hyperplasia occurs commonly, several other important molecular causes have been reported, often with overlapping clinical and biochemical features. The relative prevalence of these conditions is not known, but making a specific diagnosis can have important implications for management.

**Objective:**

The objective of the study was to investigate the clinical and molecular genetic characteristics of a nationwide cohort of children with PAI of unknown etiology.

**Design:**

A structured questionnaire was used to evaluate clinical, biochemical, and imaging data. Genetic analysis was performed using Haloplex capture and next-generation sequencing. Patients with congenital adrenal hyperplasia, adrenoleukodystrophy, autoimmune adrenal insufficiency, or obvious syndromic PAI were excluded.

**Setting:**

The study was conducted in 19 tertiary pediatric endocrinology clinics.

**Patients:**

Ninety-five children (48 females, aged 0–18 y, eight familial) with PAI of unknown etiology participated in the study.

**Results:**

A genetic diagnosis was obtained in 77 patients (81%). The range of etiologies was as follows: *MC2R* (n = 25), *NR0B1* (n = 12), *STAR* (n = 11), *CYP11A1* (n = 9), *MRAP* (n = 9), *NNT* (n = 7), *ABCD1* (n = 2), *NR5A1* (n = 1), and *AAAS* (n = 1). Recurrent mutations occurred in several genes, such as c.560delT in *MC2R*, p.R451W in *CYP11A1*, and c.IVS3ds+1delG in *MRAP*. Several important clinical and molecular insights emerged.

**Conclusion:**

This is the largest nationwide study of the molecular genetics of childhood PAI undertaken. Achieving a molecular diagnosis in more than 80% of children has important translational impact for counseling families, presymptomatic diagnosis, personalized treatment (eg, mineralocorticoid replacement), predicting comorbidities (eg, neurological, puberty/fertility), and targeting clinical genetic testing in the future.

Primary adrenal insufficiency (PAI) is a potentially life-threatening condition that requires accurate diagnosis and urgent treatment with glucocorticoid and often mineralocorticoid replacement. Because the symptoms and signs of adrenal insufficiency are often nonspecific, the diagnosis may be easily overlooked (1).

In contrast to the predominance in autoimmune etiologies in adults, most causes of PAI in childhood have an inherited, monogenic origin (1–3). Genetic causes of pediatric PAI can be classified into four major groups according to the underlying pathogenesis; 1) impaired steroidogenesis, 2) adrenal hypoplasia, 3) familial glucocorticoid deficiency (FGD) and FGD-like disorders, and 4) adrenal destruction.

Congenital adrenal hyperplasia (CYP21A2, CYP11B1, HSD3B2, CYP17A1, POR deficiencies) constitutes the largest subgroup of impaired steroidogenesis and is the most common cause of PAI in children (1, 2, 4). In contrast, there are other individually rare causes of PAI. Several genetic causes of adrenal hypoplasia (*NR0B1/*dosage-sensitive sex reversal, adrenal hypoplasia congenita critical region, on the X chromosome, gene 1 [DAX-1], *NR5A1/SF-1*, *CDKN1C* gene defects), congenital lipoid adrenal hyperplasia (*CYP11A1*, *STAR* gene defects), familial glucocorticoid deficiency (FGD) and FGD-like conditions (*MC2R* [FGD1], *MRAP* [FGD2], *STAR, MCM4*, *NNT*, *TXNRD2* gene defects) and adrenal destruction (*AIRE*, *ABCD1*, *PEX1*, *LIPA* gene defects) are now well established (5–16). However, it is also emerging that there is considerable overlap in the clinical and biochemical presentation of these conditions. For example, FGD/FGD-like conditions (*MC2R*, *MRAP*, *NNT* gene defects) can present with salt loss suggestive of adrenal hypoplasia, and alterations in *STAR* and *CYP11A1* resulting in partial loss of protein function may have a predominant FGD-like phenotype (17–20).

Establishing a specific genetic diagnosis of PAI is extremely valuable for identifying presymptomatic children who could benefit from treatment before the onset of potentially life-threatening symptoms and for counseling family members appropriately about the risk of passing the condition on to their children (1, 3, 20, 21). Knowing the genetic etiology can also help to modify treatments, such as the need for long-term mineralocorticoid replacement, and can predict potential comorbidities, such as impaired puberty or fertility and neurological dysfunction.

Next-generation sequencing (NGS) approaches are revolutionizing our ability to sequence large numbers of genes quickly and cost effectively. In this study, a custom panel-based NGS approach has been used to sequence all known PAI-associated genes in a national cohort of 95 children with PAI of unknown etiology.

## Patients and Methods

### Patients

**Figure 1.**
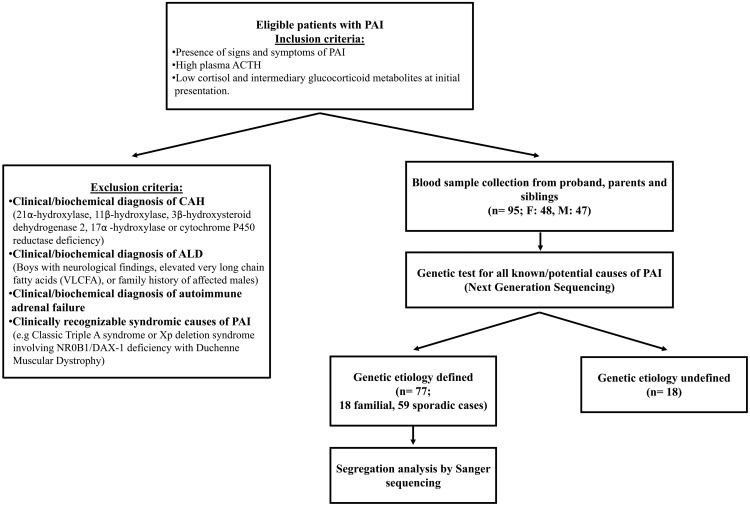
Overview of the study design, recruitment, and outcome of genetic analysis. ALD, adrenoleukodystrophy.

A pediatric cohort study was performed with PAI patients recruited from 19 pediatric endocrinology clinics in Turkey. Inclusion criteria of a PAI phenotype was defined as the presence of signs and symptoms of adrenal insufficiency together with high plasma ACTH and low serum cortisol and intermediary glucocorticoid metabolites at initial presentation. Exclusion criteria were as follows: 1) congenital adrenal hyperplasia (21α-hydroxylase, 11β-hydroxylase, 3β-hydroxysteroid dehydrogenase 2, 17α-hydroxylase, or cytochrome P450 reductase deficiencies) diagnosed by a distinctive serum steroid hormone profiles; 2) X-linked adrenoleukodystrophy in boys with neurological findings and elevated very long-chain fatty acids, or a family history of affected males with adrenoleukodystrophy; 3) clinical and biochemical evidence of autoimmune adrenal failure; and 4) known syndromic causes of PAI (specifically, classic Triple A syndrome or Xp deletion syndrome involving *NR0B1/*DAX-1 with Duchenne muscular dystrophy) (Figure 1).

All patients were assessed by a pediatric endocrinologist. A structured questionnaire was used to systematically evaluate all clinical, biochemical, and imaging data related to the diagnosis and treatment of PAI and all other relevant medical and family history. Studies were performed with the approval of the Ethics Committee of the Marmara University Faculty of Medicine (Istanbul, Turkey; B.30.2.MAR.0.01.02/AEK/108). Patients and/or parents provided written informed consent, and all studies were conducted in accordance with the principles of the Declaration of Helsinki.

A total of 95 PAI patients (48 females and 47 males) from 85 families and their unaffected siblings and parents were included (Figure 1). The most common presenting features were hyperpigmentation (94%), salt-wasting crisis/electrolyte imbalance (51%), hypoglycemia with/without convulsions (47%), vomiting/abdominal pain (26%), prolonged jaundice (24%), fatigue (18%), neonatal respiratory distress (17%), frequent infections (11%), and failure to thrive or weight loss (7%). Seven patients had 46,XY disorders of sex development (DSD). Detailed clinical findings are provided in Supplemental Table 1.

Parents of patients were consanguineous in 63 (74%) families, whereas 22 families did not report consanguinity. A total of 51 patients were on hydrocortisone treatment alone, whereas 44 were also commenced on mineralocorticoid replacement due to salt wasting, high plasma renin activity, or low aldosterone. Eight families had multiple affected siblings (six pairs, two trios, n = 18).

### Molecular analyses

#### DNA samples

Genomic DNA was extracted from whole blood of patients, parents, and available unaffected siblings using a QIAamp DNA blood maxikit (QIAGEN Inc).

### Design of targeted gene panel

A custom HaloPlex DNA target enrichment panel (Agilent Technologies Inc) was designed (SureDesign) to capture 160 known and candidate genes involved in adrenal development and function. All coding exons and 100 base pairs of intronic flanking sequence were included. The panel covered known genes potentially causing PAI, congenital adrenal hyperplasia-related genes, potential syndrome-related genes and candidate genes based on data from biochemical/biological pathways, mouse models of adrenal dysfunction, and gene expression (Supplemental Methods).

### Sequence capture and NGS

Sequence capture was performed according to the HaloPlex Target Enrichment Protocol version D.5 (Agilent Technologies Inc) for Illumina sequencing (Supplemental Methods). Patient genomic DNA aliquots (225 ng) were processed in batches of 24 samples at a time with an enrichment control DNA sample as a positive control. Sequencing was performed on a MiSeq next-generation sequencer (Illumina Inc).

### Variant analysis

Sequence alignment and variant calling were performed using SureCall (version 2.0) software (Agilent Technologies Inc). All potential disease causing variants were confirmed by PCR and Sanger sequencing. Variants in known disease genes were considered highly likely to be pathogenic if they segregated with the phenotype with an appropriate inheritance pattern within families, were determined damaging or likely damaging by several bioinformatic prediction models (Ensembl Variant Effector Predictor; SIFT; PolyPhen2; and Mutation Taster) and if they had been reported previously. In addition, novel missense changes were absent in at least 200 Turkish control samples and had a minor allele frequency less than 1:100 000 in the Exome Aggregation Consortium (ExAC) browser (ExAC; Cambridge, MA, http://exac.broadinstitute.org; accessed July 2015).

More detailed description of methods, workflows, coverage, and quality control are provided in Supplemental Methods.

## Results

A molecular genetic diagnosis was obtained in 81% children with PAI (77 of 95) using this targeted NGS approach. A total of 43 different deleterious nonsynonymous variations were detected in nine different genes (Table 1). These changes were all confirmed by Sanger sequencing and included missense mutations (n = 24), nonsense mutations (n = 7), frameshift mutations (n = 5), in-frame single codon deletions (n = 3), splice site disruptions (n = 2), and whole gene/exon deletions (n = 2). Of these variations, 22 (51%) had not been reported previously but were considered to be causative because they segregated with the phenotype in the family, they were classed as damaging or probably damaging by several bioinformatic predictions, and they were not found in the control samples or databases (Supplemental Table 2). The remaining 21 mutations have been reported (Supplemental Table 1). A molecular diagnosis was reached in all eight families with multiple affected siblings, including novel changes in five families (Table 1).

**Table 1.** Sequence Variations Detected in Our Cohort of 95 Children With Primary Adrenal Insufficiency

Gene (Chromosome)	Familial, n	Sporadic, n	Total, n	Variants	n	Consanguinity	Mineralocorticoid Treatment
*MC2R* (18p11.21)	4	21	25			22/25 (88%)	2/25 (8%)
				p.D103N	1		
				p.G116V	2		
				p.R137W	1		
				p.V142L	1		
				**p.T143S**	1		
				p.L225R	1		
				p.G226R	1		
				p.A233P	2		
				**p.C251W**	2		
				c.560delT (p.V187Afs*29)	10		
				**Deletion**	3		
*NR0B1* (Xp21.2)^a^	6^b^	6	12			3/12 (25%)	12/12 (100%)
				p.W235*	3		
				p.W236*	1		
				p.E256*	3		
				p.W291C	1		
				**p.L299R**	1		
				p.Y378*	1		
				p.C396*	1		
				p.V269del	1		
*STAR* (8p11.23)	2	9	11			8/11 (72%)	11/11 (100%)
				**p.S13P**	3		
				**p.W96C**	2		
				p.L157P	1		
				p.E169K	1		
				p.R182H	1		
				p.W250*/**p.I166M**	1		
				p.S12Afs*9	1		
				**p.K159del**	1		
*CYP11A1* (15q24.1)	2	7	9			8/9 (89%)	6/9 (67%)
				p.R451W	9		
*MRAP* (21q22.11)	2	7	9			5/9 (56%)	2/9 (22%)
				**p.L53P**	1		
				c.IVS3ds + 1insT	1		
				c.IVS3ds + 1delG	5		
				**p.K30del**	2		
*NNT* (5p12)	2	5	7			7/7 (100%)	2/7 (29%)
				**p.D178G**	1		
				**p.H370R**	1		
				c.1769dupA (p.D590Efs*29)	1		
				c.2396delC (p.P799Qfs*22)	1		
				c.127_128delTG (p.W43Vfs*2)	2		
				**Deletion (exon 2–3)**	1		
*ABCD1* (Xq28)^a^	0	2	2			0/2	1/2
				p.G512S	1		
				**p.Y547C**	1		
*NR5A1* (9q33.1)	0	1	1			0/1	1/1
				p.R92Q	1		
*AAAS* (12q13.13)	0	1	1			1/1	0/1
				p.R445*	1		
Total	**18**	**59**	**77**			**54/77 (70%)**	**37/77 (48%)**

Novel variants are marked in bold. All mutations are homozygous except for hemizygous mutations in X-linked genes and p.W250*/p.I166M in *STAR* which was compound heterozygous.

a Hemizygous mutations in X-linked genes.

b Familial cases included sibling pairs except for *NR0B1* in which two sibling trios were identified. Nucleotide position of variants is shown in Supplemental Table 1.

**Figure 2.**
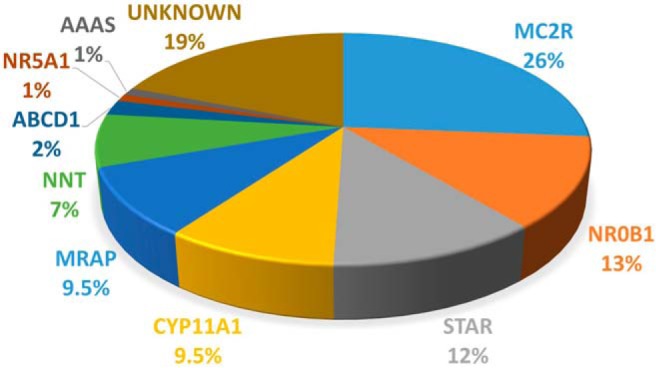
Pie chart showing the percentage of mutations in each gene in this cohort of children with PAI.

**Figure 3.**
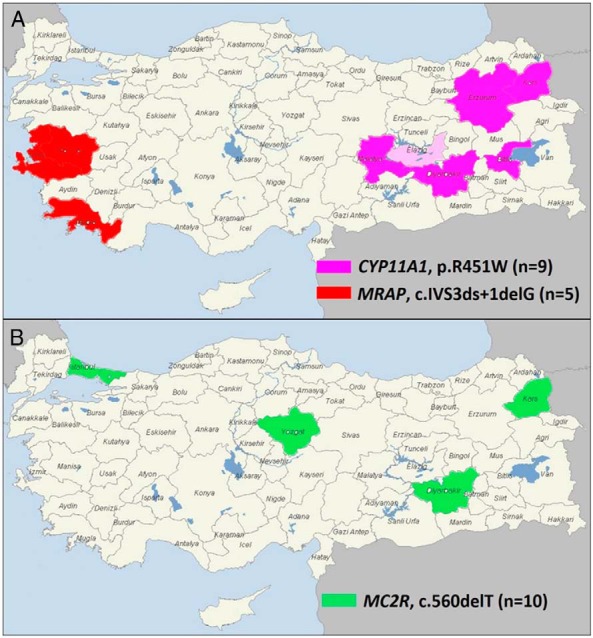
Geographical distribution of recurrent mutations identified in this study. A, The *MRAP* c.IVS3ds+1delG mutation was identified mainly in patients from west Turkey, whereas the *CYP11A1* p.R451W mutation was found in patients who originated from east Turkey. The first report of the *CYP11A1* p.R451W mutation was in a family from Germany who were originally from Elazig (shown in light pink) (20). B, The *MC2R* c.560delT mutation showed a wider distribution most likely reflecting migration from the east to west of Turkey and has been described previously in a family from northern Iran (17).

The range of genetic etiologies found in this cohort were as follows: *MC2R* (n = 25), *NR0B1* (n = 12), *STAR* (n = 11), *CYP11A1* (n = 9), *MRAP* (n = 9), *NNT* (n = 7), *ABCD1* (n = 2), *NR5A1* (n = 1), and *AAAS* (n = 1) (Figure 2). Most patients were homozygous for recessive changes (62 of 77, 80%), one patient carried compound heterozygous changes (1 of 77, 1.3%), and 14 patients had hemizygous mutations in X-linked genes (*NR0B1*, *ABCD1*) (14 of 77, 18%). As expected, consanguinity rates were much higher in families of patients harboring mutations in recessive genes (51 of 63, 81%) compared with X-linked genes (3 of 14, 21%; *P* < .0001) (Table 1). Recurrent mutations were detected in several genes, such as c.560delT in *MC2R* (10 patients from nine unrelated families), p.R451W in *CYP11A1* (nine patients from eight unrelated families), c.IVS3ds+1delG in *MRAP* (five patients from five unrelated families), and p.S13P in *STAR* (three patients from two unrelated families). Geographical hot spots were found for the p.R451W *CYP11A1* mutation in eastern Turkey and for the c.IVS3ds+1delG *MRAP* mutation in the west (Figure 3, A and B).

**Figure 4.**
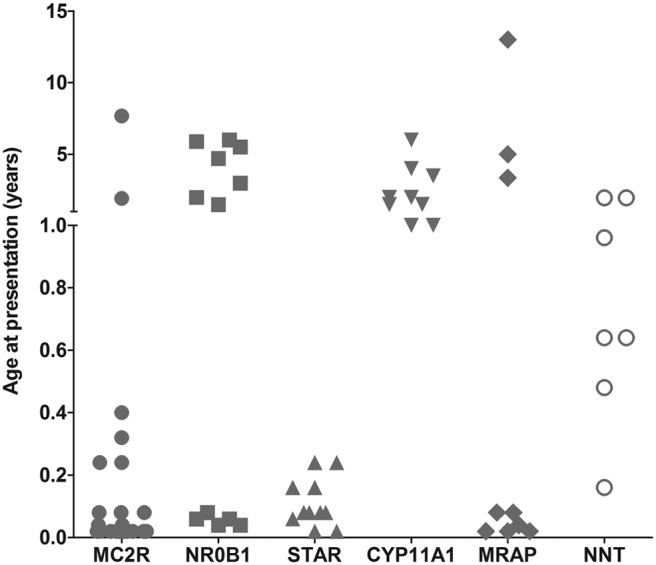
Age at presentation of the patients with PAI.

Although there was considerable overlap in the clinical and biochemical features of children within this cohort (Supplemental Table 1), several notable findings have emerged. For example, most patients with *MC2R*, *MRAP*, and *STAR* mutations presented in the first weeks or months of life, whereas children with the p.R451W mutation in *CYP11A1* presented in early childhood (1–6 y) (Figure 4). Children with nicotinamide nucleotide transhydrogenase (*NNT*) changes presented at different ages in the first 2 years, whereas boys with *NR0B1* (DAX-1) mutations had a bimodal pattern, presenting either in the first month of life or else after 18 months.

Most children had extremely high ACTH levels at diagnosis and almost all children, even babies, were clinically hyperpigmented. Hypoglycemia was a frequent finding, and hypoglycemic convulsions at presentation were more common in children with *MC2R* (22 of 25, 88%) and *MRAP* (five of nine, 56%) mutations than in children with an alternative diagnosis (5 of 43, 12%) (*P* < .0001). Salt-wasting states requiring mineralocorticoid replacement occurred in all children with *NR0B1* (DAX-1) and *STAR* mutations and in most children with the *CYP11A1* p.R451W change (six of nine, 66%) (Table 1). Fewer children with *NNT* (two of seven, 28%), *MRAP* (two of nine, 22%), and *MC2R* (2 of 25, 8%) mutations required mineralocorticoid replacement, although four additional children with *MC2R* defects had transient hyponatremia (sodium 117–133 mmol/L) that resolved without fludrocortisone treatment (Supplemental Table 1). Adrenal imaging was generally uninformative, showing normal sized or hypoplastic glands for most of these diagnoses, including many children with *STAR* mutations (congenital lipoid adrenal hyperplasia) in which enlarged adrenals are reported (22).

Additional clinical features were seen in many children in this cohort, such as altered growth, neuromotor delay, learning difficulties, and cardiac defects (Supplemental Table 1). Abnormalities in thyroid function such as subclinical hypothyroidism were common (n = 20, 26%), whereas thyroglossal cysts (n = 2), primary hypothyroidism (n = 2), and transient hypothyroidism (n = 1) were also found. DSD occurred in all six 46,XY infants with *STAR* mutations. Five of these children were phenotypic females who presented with a salt-losing crisis, and in two cases a karyotype was not available prior to this genetic analysis. The one boy with severe hypospadias due to *STAR* deficiency developed hypergonadotropic hypogonadism in puberty and needed T replacement. One of the four boys with the p.R451W mutation in *CYP11A1* had a small penis and cryptorchidism. One boy with a *NR0B1* mutation and X-linked adrenal hypoplasia congenita (AHC) had macrophallia and another one had cryptorchidism. Coincidental hypospadias and unilateral cryptorchidism was found in one boy with disruption of *MC2R*.

Stature was variable in the 25 children with *MC2R* mutations with seven children (28%) having tall stature (>2 SD score or two percentile lines above parental target height), four having short stature (<−2 SD score) and the rest being within the normal range. There was no history of preterm birth associated with *CYP11A1* mutations, but there were reports of multiple stillbirths in three families in which a child had severe disruption of *MC2R* (deletion or c.560delT frameshift). Despite the range of neurological and other features seen, the two boys with mutations in *ABCD1* (causing X-linked adrenoleukodystrophy) and one boy with disruption of *AAAS* (causing Triple A syndrome) appeared to have adrenal only phenotypes and would not have been diagnosed without genetic testing.

## Discussion

The past 20 years has seen significant progress in our understanding of the genetic causes of childhood PAI. However, it is unclear how much these genes contribute to pediatric adrenal disease in the clinical setting as most reports have focused on specific categories of adrenal disease such as FGD or adrenal hypoplasia (6, 9).

In this study, an unbiased nationwide cohort of almost 100 children with PAI was recruited from 19 pediatric endocrinology centers across Turkey, and a molecular diagnosis was reached in more than 80% of the children. This represents the largest clinical cohort of children with this rare condition assembled. Because children could die from convulsions, respiratory distress, or salt-losing crises before reaching the hospital or get misdiagnosed with sepsis, the condition may be underdiagnosed. In fact, a history of unexplained death in infancy or childhood in the extended family was common in many of those questioned.

Targeted panel-based capture and high-throughput sequencing proved very effective in reaching a molecular diagnosis in a relatively quick and comprehensive manner. A total of 43 known and novel mutations in nine genes were discovered in 77 patients, with 73 of 77 mutations (95%) occurring in six genes (*MC2R*, *NR0B1*, *STAR*, *CYP11A1*, *MRAP*, and *NNT*). Several recurrent mutations were discovered, which likely represent founder effects. Some of these are localized to certain geographical areas (eg, p.R451W in *CYP11A1* in eastern Turkey, c.IVS3ds+1delG in *MRAP* in western Turkey), which could lead to focused cost-effective clinical genetic testing for patients and families at risk of adrenal insufficiency in these regions (Figure 3A). Other recurrent changes, such as the c.560delT in *MC2R* showed more diverse geographical distribution across the country, possibly reflecting migration toward the west (Figure 3B). Indeed, this *MC2R* mutation has been reported previously in the father of two siblings with FGD1 who originated from northern Iran, close to the Turkish border (17). Sanger sequencing for just the three changes in *MC2R*, *CYP11A1*, and *MRAP* would have diagnosed 22 of 85 different families recruited in this nationwide cohort (26%), which could represent a cost-effective approach to first-line clinical genetic analysis. Of note, no mutations were found in *MCM4* or*TXNRD2*, which to date have been described only in Irish Traveler and Kashmiri families, respectively (12, 14, 23)

In addition to defining the population genetics of PAI, this study has provided some useful clinical and novel molecular insight into several of these specific conditions.

Mutations in *MC2R* (encoding the ACTH receptor) are well established as the cause of FGD1, and approximately 40 different missense changes have been reported (9). Children typically present with hypoglycemia or hyperpigmentation in early infancy or in childhood, but complete loss of function mutations are rare (17, 24). Although diverse missense mutations were common in our cohort, severely disruptive changes in *MC2R* were found in more than half of the children (13 of 25), all of whom presented in the first 6 months of life with hypoglycemic convulsions, respiratory distress, or both. In addition to the c.560delT mutation discussed above, the first complete deletions of the *MC2R* locus were found in three patients. Although ACTH plays a facilitative role in supporting mineralocorticoid release, children with FGD1 do not typically have salt loss. However, transient hyponatremia has been reported in several children with severe disruption of the receptor, sometimes leading to a misdiagnosis of adrenal hypoplasia (17, 25). Similar observations were seen in this cohort, with 5 of 25 children having evidence of hyponatremia (sodium 117–133 mmol/L) but only two of them receiving long-term fludrocortisone replacement. On the basis of the genetic diagnosis, it is likely the need for this treatment can be reviewed. The significance of recurrent stillbirths in three families with severe loss of *MC2R* function is unclear. Although tall stature at diagnosis has been suggested for *MC2R* mutations, any effects on growth are difficult to interpret because most of our cohort were diagnosed and treated in infancy (26).

Hemizygous mutations in *NR0B1* (encoding the nuclear receptor DAX-1) were found in 12 boys, including in two families in which three sons were affected. Mutations in *NR0B1* cause X-linked AHC and more than 100 different mutations are reported (5). Most are frameshift or nonsense mutations that disrupt protein function, with a clustering of missense changes in three regions of the ligand-like binding domain (27). The three missense changes identified here (p.V269del, p.W291C, p.L299R) are located in a loop region of helix 3 to helix 5 that interacts with NR5A nuclear receptors (such as steroidogenic factor-1) (28). The novel p.L299 lies adjacent to the frequently mutated p.A300 residue, whereas codons p.V269 and p.W291 have been mutated previously in non-Turkish pedigree (5). All DAX-1-deficient boys presented with salt loss and showed a bimodal distribution pattern of age at presentation as reported previously (Figure 4) (29). Although currently preadolescent, they are likely to develop hypogonadotropic hypogonadism and infertility as part of their condition, so establishing the diagnosis in childhood will help plan endocrine management of puberty, counseling, and potential assisted reproduction (30). Macrophallia in infancy, found in one boy, is emerging as a rare feature of X-linked AHC (31).

True CYP11A1 deficiency (encoding P450 side chain cleavage [P450scc]) is a relatively recently established endocrine condition because it was thought that disruption of this enzyme, which facilitates the first three steps in conversion of cholesterol to pregnenolone, would be incompatible with fetal survival in humans (4). However, several children with this condition are now reported (32, 33). Severe loss of CYP11A1/P450scc function is usually associated with severe salt-losing adrenal failure in the neonatal period and a female phenotype in 46,XY children (46,XY DSD). Milder changes can present later with adrenal insufficiency and a history of hypospadias in boys (34, 35). The p.R451W variant found in all our patients was described recently in two brothers of Turkish origin residing in Germany who had normal genitalia and childhood-onset PAI (20). Their family originated from the Elazig region of Eastern Anatolia, a province bordering two regions where our patients lived (Figure 3A). The p.R451W mutation disrupts hydrogen bonds with residues F428 and Y449 in the K-L loop of the enzyme, causing partial loss of function consistent with the late presentation and normal genitalia in three of the four boys (20). Nevertheless, all children will need careful follow-up through puberty and into adult life to monitor sex hormone production and fertility. These findings confirm that mild loss of CYP11A1/P450scc function can present with isolated adrenal insufficiency and normal male genitalia, similar to mild loss of function of STAR (18–20).

Defects in *STAR* (encoding steroidogenic acute regulatory protein) disrupt the transport of cholesterol into mitochondria and classically lead to congenital lipoid adrenal hyperplasia (4, 22). A range of mutations were found, including recurrent p.S13P changes in three patients including two siblings. This is by far the most aminoterminal STAR mutation reported to date and affects the mitochondrial leader that is involved in targeting and localizing STAR to the outer mitochondrial membrane (36). Classic congenital lipoid adrenal hyperplasia is associated with severe salt-losing adrenal failure and 46,XY DSD due to a block in both adrenal and testicular steroidogenesis. The two-hit hypothesis suggests that after an initial reduction in steroid production and increased ACTH drive, accumulation of intracellular lipid has a secondary toxic effect on cells (22). Interestingly, nine of the children presented between 3 weeks and 3 months of age, consistent with the two-hit hypothesis, whereas only two presented in the first week (22). Two girls were found to have 46,XY DSD only after genetic analysis revealed *STAR* deficiency, highlighting the importance of obtaining a karyotype in phenotypic girls with salt-losing adrenal failure. As with CYP11A1/P450scc deficiency, these children all need endocrine follow-up for life to monitor puberty and ovarian function in 46,XX girls and for puberty induction and sex steroid replacement in 46,XY DSD. Indeed, the one patient with hypospadias raised male is now showing evidence of hypergonadotropic hypogonadism in adolescence.

Mutations in *MRAP* (encoding melanocortin 2 receptor-associated protein) and *NNT* (encoding nicotinamide nucleotide transhydrogenase) cause adrenal insufficiency without other features and can only be diagnosed by genetic analysis, as shown here. MRAP is essential for trafficking the ACTH receptor (melanocortin 2 receptor) to the cell membrane, and mutations causing FGD2 were first described in 2005 (9, 10, 24). The intron 3 splice site is especially vulnerable, often resulting in early-onset adrenal insufficiency. Patients with novel aminoterminal point mutations (p.K30del, p.L53P) presented unusually late (3.5–13 y) (Figure 4), consistent with reports of children with p.V26A and p.Y59D changes (37).

*NNT* mutations affect cellular oxidation and were first decribed in 2012; approximately 20 children have been reported to date (9, 13, 38). The six novel changes found here include familial homozygous deletions of exons 2–3 and homozygous missense mutations in the mitochondrial matrix region (p.D178G, p.H370R). These findings confirm the importance of NNT for adrenal function in an independent cohort. Unlike in other studies, compound heterozygous mutations were not found (13).

Heterozygous mutations in *NR5A1* (encoding the nuclear receptor steroidogenic factor-1) usually cause 46,XY DSD or primary ovarian insufficiency (5). Adrenal insufficiency is extremely rare and has been reported only once, in a 46,XX girl (39). The homozygous p.R92Q mutation found here in a 46,XX girl with early-onset PAI is fascinating because the same homozygous change was reported in a 46,XY phenotypic female with adrenal failure from central Turkey in 2002 (40). This finding reinforces the importance of the A-box motif of steroidogenic factor 1 in monomeric binding to DNA and provides conclusive evidence that severe disruption of steroidogenic factor 1 can cause adrenal insufficiency in humans.

Finally, mutations identified in *AAAS* (typically causing Triple A syndrome: achalasia, alacrima, Addison disease) and *ABCD1* (typically causing X-linked adrenoleukodystrophy) in three children without other features shows how genetic screening can identify adrenal-only phenotypes in young people who may be at risk of developing other symptoms in later life (11, 15).

This nationwide cohort study of high-throughput genetic screening of children with rare causes of PAI has provided many novel and supportive clinical and molecular insights and has significant impact on the management of these patients and their families. New genetic technologies are a powerful tool in defining population genetics of rare conditions and will allow more focused clinical genetic screening programs to be established.

## References

[1] Hsieh S White PC Presentation of primary adrenal insufficiency in childhood. J Clin Endocrinol Metab. 2011;96:E925–E928. 21470994 10.1210/jc.2011-0015

[2] Perry R Kecha O Paquette J Huot C Van Vliet G Deal C Primary adrenal insufficiency in children: twenty years‘ experience at the Sainte-Justine Hospital, Montreal. J Clin Endocrinol Metab. 2005;90:3243–3250. 15811934 10.1210/jc.2004-0016

[3] Malikova J Flück CE Novel insight into etiology, diagnosis and management of primary adrenal insufficiency. Horm Res Paediatr. 2014;82:145–157. 25096886 10.1159/000363107

[4] Miller WL Auchus RJ The molecular biology, biochemistry, and physiology of human steroidogenesis and its disorders. Endocr Rev. 2011;32:81–151. 21051590 10.1210/er.2010-0013 PMC3365799

[5] Suntharalingham JP Buonocore F Duncan AJ Achermann JC DAX-1 (NR0B1) and steroidogenic factor-1 (SF-1, NR5A1) in human disease. Best Pract Res Clin Endocrinol Metab. 2015;29:607–619. 26303087 10.1016/j.beem.2015.07.004 PMC5159745

[6] Lin L Gu WX Ozisik G Analysis of DAX1 (NR0B1) and steroidogenic factor-1 (NR5A1) in children and adults with primary adrenal failure: ten years' experience. J Clin Endocrinol Metab. 2006;91:3048–3054. 16684822 10.1210/jc.2006-0603 PMC1865080

[7] Arboleda VA Lee H Parnaik R Mutations in the PCNA-binding domain of CDKN1C cause IMAGe syndrome. Nat Genet. 2012;44:788–792. 22634751 10.1038/ng.2275 PMC3386373

[8] Meimaridou E Hughes CR Kowalczyk J Chan LF Clark AJ Metherell LA ACTH resistance: genes and mechanisms. Endocr Dev. 2013;24:57–66. 23392095 10.1159/000342504

[9] Meimaridou E Hughes CR Kowalczyk J Familial glucocorticoid deficiency: New genes and mechanisms. Mol Cell Endocrinol. 2013;371:195–200. 23279877 10.1016/j.mce.2012.12.010

[10] Metherell LA Chapple JP Cooray S Mutations in MRAP, encoding a new interacting partner of the ACTH receptor, cause familial glucocorticoid deficiency type 2. Nat Genet. 2005;37:166–170. 15654338 10.1038/ng1501

[11] Dumic M Barišic N Kusec V Long-term clinical follow-up and molecular genetic findings in eight patients with triple A syndrome. Eur J Pediatr. 2012;171:1453–1459. 22538409 10.1007/s00431-012-1745-1

[12] Hughes CR Guasti L Meimaridou E MCM4 mutation causes adrenal failure, short stature, and natural killer cell deficiency in humans. J Clin Invest. 2012;122:814–820. 22354170 10.1172/JCI60224 PMC3287227

[13] Meimaridou E Kowalczyk J Guasti L Mutations in NNT encoding nicotinamide nucleotide transhydrogenase cause familial glucocorticoid deficiency. Nat Genet. 2012;44:740–742. 22634753 10.1038/ng.2299 PMC3386896

[14] Prasad R Chan LF Hughes CR Thioredoxin reductase 2 (TXNRD2) mutation associated with familial glucocorticoid deficiency (FGD). J Clin Endocrinol Metab. 2014;99:E1556–E1563. 24601690 10.1210/jc.2013-3844 PMC4207928

[15] Engelen M Kemp S de Visser M X-linked adrenoleukodystrophy (X-ALD): clinical presentation and guidelines for diagnosis, follow-up and management. Orphanet J Rare Dis. 2012;7:51. 22889154 10.1186/1750-1172-7-51 PMC3503704

[16] Berendse K Engelen M Linthorst GE van Trotsenburg AS Poll-The BT High prevalence of primary adrenal insufficiency in Zellweger spectrum disorders. Orphanet J Rare Dis. 2014;9:133. 25179809 10.1186/s13023-014-0133-5 PMC4164755

[17] Lin L Hindmarsh PC Metherell LA Severe loss-of-function mutations in the adrenocorticotropin receptor (ACTHR, MC2R) can be found in patients diagnosed with salt-losing adrenal hypoplasia. Clin Endocrinol (Oxf). 2007;66:205–210. 17223989 10.1111/j.1365-2265.2006.02709.x PMC1859977

[18] Baker BY Lin L Kim CJ Nonclassic congenital lipoid adrenal hyperplasia: a new disorder of the steroidogenic acute regulatory protein with very late presentation and normal male genitalia. J Clin Endocrinol Metab. 2006;91:4781–4785. 16968793 10.1210/jc.2006-1565 PMC1865081

[19] Metherell LA Naville D Halaby G Nonclassic lipoid congenital adrenal hyperplasia masquerading as familial glucocorticoid deficiency. J Clin Endocrinol Metab. 2009;94:3865–3871. 19773404 10.1210/jc.2009-0467 PMC2860769

[20] Parajes S Kamrath C Rose IT A novel entity of clinically isolated adrenal insufficiency caused by a partially inactivating mutation of the gene encoding for P450 side chain cleavage enzyme (CYP11A1). J Clin Endocrinol Metab. 2011;96:E1798–1806. 21880796 10.1210/jc.2011-1277

[21] Achermann JC Silverman BL Habiby RL Jameson JL Presymptomatic diagnosis of X-linked adrenal hypoplasia congenita by analysis of DAX1. J Pediatr. 2000;137:878–881. 11113848 10.1067/mpd.2000.108567

[22] Bose HS Sugawara T Strauss JF 3rd Miller WL International Congenital Lipoid Adrenal Hyperplasia Consortium. The pathophysiology and genetics of congenital lipoid adrenal hyperplasia. N Engl J Med. 1996;335:1870–1878. 8948562 10.1056/NEJM199612193352503

[23] Gineau L Cognet C Kara N Partial MCM4 deficiency in patients with growth retardation, adrenal insufficiency, and natural killer cell deficiency. J Clin Invest. 2012;122:821–832. 22354167 10.1172/JCI61014 PMC3287233

[24] Chung TT Chan LF Metherell LA Clark AJ Phenotypic characteristics of familial glucocorticoid deficiency (FGD) type 1 and 2. Clin Endocrinol (Oxf). 2010;72:589–594. 19558534 10.1111/j.1365-2265.2009.03663.x PMC2855830

[25] Chan LF Metherell LA Krude H Homozygous nonsense and frameshift mutations of the ACTH receptor in children with familial glucocorticoid deficiency (FGD) are not associated with long-term mineralocorticoid deficiency. Clin Endocrinol (Oxf). 2009;71:171–175. 19170705 10.1111/j.1365-2265.2008.03511.x PMC2728896

[26] Yeh JK Evans JF Niu QT Aloia JF A possible role for melanocortin peptides in longitudinal growth. J Endocrinol. 2006;191:677–686. 17170224 10.1677/joe.1.06729

[27] Achermann JC Ito M Silverman BL Missense mutations cluster within the carboxyl-terminal region of DAX-1 and impair transcriptional repression. J Clin Endocrinol Metab. 2001;86:3171–3175. 11443184 10.1210/jcem.86.7.7660

[28] Sablin EP Woods A Krylova IN Hwang P Ingraham HA Fletterick RJ The structure of corepressor Dax-1 bound to its target nuclear receptor LRH-1. Proc Natl Acad Sci USA. 2008;105:18390–18395. 19015525 10.1073/pnas.0808936105 PMC2587556

[29] Reutens AT Achermann JC Ito M Clinical and functional effects of mutations in the DAX-1 gene in patients with adrenal hypoplasia congenita. J Clin Endocrinol Metab. 1999;84:504–511. 10022408 10.1210/jcem.84.2.5468

[30] Frapsauce C Ravel C Legendre M Birth after TESE-ICSI in a man with hypogonadotropic hypogonadism and congenital adrenal hypoplasia linked to a DAX-1 (NR0B1) mutation. Hum Reprod. 2011;26:724–728. 21227944 10.1093/humrep/deq372 PMC3037794

[31] Landau Z Hanukoglu A Sack J Clinical and genetic heterogeneity of congenital adrenal hypoplasia due to NR0B1 gene mutations. Clin Endocrinol (Oxf). 2010;72:448–454. 19508677 10.1111/j.1365-2265.2009.03652.x

[32] Hiort O Holterhus PM Werner R Homozygous disruption of P450 side-chain cleavage (CYP11A1) is associated with prematurity, complete 46,XY sex reversal, and severe adrenal failure. J Clin Endocrinol Metab. 2005;90:538–541. 15507506 10.1210/jc.2004-1059

[33] Kim CJ Lin L Huang N Severe combined adrenal and gonadal deficiency caused by novel mutations in the cholesterol side chain cleavage enzyme, P450scc. J Clin Endocrinol Metab. 2008;93:696–702. 18182448 10.1210/jc.2007-2330 PMC2266942

[34] Rubtsov P Karmanov M Sverdlova P Spirin P Tiulpakov A A novel homozygous mutation in CYP11A1 gene is associated with late-onset adrenal insufficiency and hypospadias in a 46,XY patient. J Clin Endocrinol Metab. 2009;94:936–939. 19116240 10.1210/jc.2008-1118

[35] Tee MK Abramsohn M Loewenthal N Varied clinical presentations of seven patients with mutations in CYP11A1 encoding the cholesterol side-chain cleavage enzyme, P450scc. J Clin Endocrinol Metab. 2013;98:713–720. 23337730 10.1210/jc.2012-2828 PMC3565115

[36] Miller WL Bose HS Early steps in steroidogenesis: intracellular cholesterol trafficking. J Lipid Res. 2011;52:2111–2135. 21976778 10.1194/jlr.R016675 PMC3283258

[37] Hughes CR Chung TT Habeb AM Kelestimur F Clark AJ Metherell LA Missense mutations in the melanocortin 2 receptor accessory protein that lead to late onset familial glucocorticoid deficiency type 2. J Clin Endocrinol Metab. 2010;95:3497–3501. 20427498 10.1210/jc.2009-2731

[38] Novoselova TV Rath SR Carpenter K NNT pseudoexon activation as a novel mechanism for disease in two siblings with familial glucocorticoid deficiency. J Clin Endocrinol Metab. 2015;100:E350–E354. 25459914 10.1210/jc.2014-3641 PMC4318891

[39] Biason-Lauber A Schoenle EJ Apparently normal ovarian differentiation in a prepubertal girl with transcriptionally inactive steroidogenic factor 1 (NR5A1/SF-1) and adrenocortical insufficiency. Am J Hum Genet. 2000;67:1563–1568. 11038323 10.1086/316893 PMC1287931

[40] Achermann JC Ozisik G Ito M Gonadal determination and adrenal development are regulated by the orphan nuclear receptor steroidogenic factor-1, in a dose-dependent manner. J Clin Endocrinol Metab. 2002;87:1829–1833. 11932325 10.1210/jcem.87.4.8376

